# Ameliorative Role of Cerium Oxide Nanoparticles Against Fipronil Impact on Brain Function, Oxidative Stress, and Apoptotic Cascades in Albino Rats

**DOI:** 10.3389/fnins.2021.651471

**Published:** 2021-05-14

**Authors:** Norhan Elshony, Atef M. K. Nassar, Yasser S. El-Sayed, Dalia Samak, Ahmed Noreldin, Lamiaa Wasef, Hamida Saleh, Yaser H. A. Elewa, Shereen E. Tawfeek, Abdullah A. Saati, Gaber El-Saber Batiha, Michał Tomczyk, Masakazu Umezawa, Hazem M. Shaheen

**Affiliations:** ^1^Department of Pharmacology and Therapeutics, Faculty of Veterinary Medicine, Damanhour University, Damanhour, Egypt; ^2^Department of Plant Protection, Faculty of Agriculture, Damanhour University, Damanhour, Egypt; ^3^Department of Veterinary Forensic Medicine and Toxicology, Faculty of Veterinary Medicine, Damanhour University, Damanhour, Egypt; ^4^Department of Histology and Cytology, Faculty of Veterinary Medicine, Damanhour University, Damanhour, Egypt; ^5^Department of Histology and Cytology, Faculty of Veterinary Medicine, Zagazig University, Zagazig, Egypt; ^6^Laboratory of Anatomy, Department of Biomedical Sciences, Graduate School of Veterinary Medicine, Hokkaido University, Sapporo, Japan; ^7^Department of Human Anatomy and Embryology, Faculty of Medicine, Zagazig University, Zagazig, Egypt; ^8^Department of Anatomy, College of Medicine, Jouf University, Sakaka, Saudi Arabia; ^9^Department of Community Medicine and Pilgrims Healthcare, Faculty of Medicine, Umm Al-Qura University, Makkah, Saudi Arabia; ^10^Department of Pharmacognosy, Faculty of Pharmacy, Medical University of Białystok, Białystok, Poland; ^11^Department of Materials Science and Technology, Faculty of Industrial Science and Technology Soga Laboratory, Tokyo University of Science, Tokyo, Japan

**Keywords:** fipronil, cerium oxide nanoparticles, oxidative stress, neurotoxicity, apoptotic cascades

## Abstract

Fipronil (FIP) is an N-phenylpyrazole insecticide that is used extensively in public health and agriculture against a wide range of pests. Exposure to FIP is linked to negative health outcomes in humans and animals including promoting neuronal cell injury, which results in apoptosis through the production of reactive oxygen species (ROS). Therefore, the purpose of the current study was to investigate the neuroprotective effects of cerium oxide nanoparticles (CeNPs) on neuronal dysfunction induced by FIP in albino rats. Male rats were randomly classified into four groups: control, FIP (5 mg/kg bwt), CeNPs (35 mg/kg bwt), and FIP + CeNPs (5 (FIP) + 35 (CeNPs) mg/kg bwt), which were treated orally once daily for 28 consecutive days. Brain antioxidant parameters, histopathology, and mRNA expression of genes related to brain function were evaluated. The results revealed oxidative damage to brain tissues in FIP-treated rats indicated by the elevated levels of malondialdehyde (MDA) and nitric oxide (NO) levels and reduced activities of antioxidant enzymes such as superoxide dismutase (SOD) and glutathione peroxidase (GPx). On the other hand, the FIP’s group that was treated with CeNPs showed decrease in MDA and NO levels and increase in SOD and GPx enzymes activity. Besides, FIP-treated rats showed decreased butyrylcholinesterase (BuChE) activity in comparison to the FIP + CeNPs group. Moreover, FIP caused up-regulation of the expression of neuron-specific enolase (NSE), caspase-3, and glial fibrillary acidic protein (GFAP) but down-regulation of B-cell lymphoma-2 (BCL-2) expression. But the FIP + CeNPs group significantly down-regulated the GFAP, NSE, and caspase-3 and up-regulated the gene expression of BCL-2. Additionally, the FIP-treated group of rats had clear degenerative lesions in brain tissue that was reversed to nearly normal cerebral architecture by the FIP + CeNPs treatment. Immunohistochemical examination of brain tissues of rats-treated with FIP showed abundant ionized calcium-binding adaptor molecule 1 (Iba-1) microglia and caspase-3 and apoptotic cells with nearly negative calbindin and synaptophysin reaction, which were countered by FIP + CeNPs treatment that revealed a critical decrease in caspase-3, Iba-1 reaction with a strong calbindin positive reaction in most of the Purkinje cells and strong synaptophysin reaction in the cerebrum and cerebellum tissues. Based on reported results herein, CeNPs treatment might counteract the neurotoxic effect of FIP pesticide via an antioxidant-mediated mechanism.

## Introduction

Fipronil (FIP; 5-amino-1-(2,6-dichloro-α,α,α-trifluoro-*p*-tolyl)-trifluoromethylsulfinyl pyrazole-3-carbonitrile) is a wide range N-phenylpyrazole insecticide that is extensively used around the world toward the management of a wide spectrum of insects, indoors pests, agricultural pests, and ectoparasitic in veterinary clinical field (Jennings et al., 2002; Bonneau et al., 2015; McMahen et al., 2015; Mossa et al., 2015). Concerns about the side effects of FIP on public health are increasing due to its widespread use commercially and domestically (Tingle et al., 2003). In case of long-term exposure to FIP, it might result in serious adverse effects to humans, such as weakness, vertigo, nausea, and headache (Chodorowski and Sein Anand, 2004). FIP has been shown to cause transient toxic symptoms as neurological dysfunction and neurotoxic manifestations in rodents (Mohamed et al., 2004; Szegedi et al., 2005) as a result of disruption of the binding of gamma-aminobutyric acid (GABA) to its receptor resulting in the uncontrolled central nervous system (CNS) as convulsion, hyperexcitation, and death (Park et al., 2016a). It was reported that FIP induced neurotoxicity through the initiation of oxidative stress and mitochondrial damages (Seydi et al., 2021). It caused oxidative stress and cellular DNA deterioration in the cell culture of rats’ pheochromocytoma (Lassiter et al., 2009). Moreover, FIP triggered neuronal cell death and induced apoptosis that was mediated primarily by the generation of reactive oxygen species (ROS) and activation of mitogen-activated protein kinase (MAPK) members followed by activation of the intrinsic apoptotic pathway (Ki et al., 2012). The excessive generation of ROS could affect the permeability of the mitochondrial membrane decreasing its potential and finally inhibit the pro-survival gene expression of for example the B-cell lymphoma-2 (BCL-2), which subsequently contributes to cellular apoptosis due to the activation of the caspase cascade (Orrenius et al., 2007). The evolution of nanotechnology and its application in medicine has opened a new era in the diagnosis and treatment of several health issues (Engin et al., 2017; Taghizadehghalehjoughi et al., 2018). Therefore, to counteract the hazardous effects of pesticides, several materials were used including nano-based materials. Cerium oxide nanoparticles’ (CeNPs) are among the most crucial metal-oxide nanoparticles, which play a technologically important role not only in synthesizing different industrial materials including polishing materials in the glass and optics industry, oxygen sensors, and ultraviolet filters (Wasef et al., 2021) but also in biological applications. For example, the antioxidant activity that is based on the ratio of Ce^3+^/Ce^4+^ on the surface of the CeNPs structure works as catalase mimetic activity, hydroxyl scavenging property (Dowding et al., 2013), nitric oxide scavenging property, and superoxide dismutase mimetic activity (Xu and Qu, 2014). CeNPs might be involved in enhancing the performance of antioxidants and stimulating cell proliferation through the reduction of intracellular levels of ROS by modulating the expression level of the major antioxidant enzymes (Hosseini et al., 2019). CeNPs are an efficient neuroprotective agents against certain neurodegenerative disorders, as 6-hydroxydopamine (6-OHDA)-induced Parkinsonian rats (Hegazy et al., 2017). Moreover, laboratory experiments documented that nanoceria protect primary spinal cord neurons and primary cortical neurons from oxidative stress (Das et al., 2007; Dowding et al., 2014), reduced apoptosis in photoreceptor cells (Kong et al., 2011), and endothelial cells (Chen et al., 2013) by modulating the apoptotic pathways. Also, CeNPs showed a promising potential in diverse disorders, such as cerebral ischemic stroke (CIS), cancer, neurodegenerative, and inflammatory diseases (Zhou et al., 2016). The aim of this research was, therefore, to examine the neuroprotective activity of CeNPs on neurotoxicity caused by FIP in albino rats.

## Materials and Methods

### Chemicals and Reagents

Cerium oxide nanopowder (CAS Number: 1306-38-3) was purchased from Sigma-Aldrich Co., United States (Cat. # 544841-5G). The CeNPs was suspended in demineralized water at a 35 mg/kg bwt concentration. FIP solution (1/20 LD_50_: 5 mg/kg bwt) was prepared by dissolving the commercial product FIPROGENT^®^ 80%WG (AgroInvest, Cairo, Egypt) in demineralized water.

### Characterization of CeNPs

Morphology and size of CeNPs were characterized using the Transmission Electron Microscopic (TEM) (JOEL, model JSM 5300, Japan). Combination of bright-field imaging at increasing magnification and off diffraction modes were used to disclose the form and size of CeNPs as a suspension in water (1/100) that was directly deposited on the film grid and observed after being dry. Also, the X-ray diffraction patterns of nanoparticles of CeNPs powder were obtained by a D/max-rA diffractometer at CuK radiation (40 kV, 80 mA) and samples were scanned at 4°/min rate.

### Animals and Study Procedures

Twenty-eight adult male albino rats (*Rattus norvegicus*) weighing 90 ± 10 g were purchased from the animal house at Faculty of Agriculture, Alexandria University, Egypt. The animals were housed in a pathogen-free environment with controlled humidity, temperature (22°C), and a 12 h light/dark cycle. The animal experiments were performed according to the Laboratory Animals of the National Institutes of Health (NIH) Care and Use Guidelines (Albus, 2012) and the study protocol was approved by the ethical committee at Damanhour University, Egypt (DMU-2019-0023). Two weeks before when the experiment was conducted, the animals were allowed to acclimatize to the testing facility condition. The rats were provided water and a balanced diet *ad libitum* under restricted hygienic conditions. They were caged into four groups each of 7 rats (*n* = 7): control, FIP (5 mg/kg/day (Badgujar et al., 2015), CeNPs (35 mg/kg/day (Choi et al., 2015; Park et al., 2018), and CeNPs + FIP (35 + 5 mg/kg/day) for 28 days by the gastric tube. Rats were kept under observation all over the experimental period for any abnormal behavior or clinical signs.

### Tissue Preparation for Biochemical Tests

On the 29th day of the experiment, all rats were prohibited from feeding overnight, weighed individually, and euthanized using an anesthesia system containing diethyl ether. The brain was excised, rinsed in physiological saline (NaCl 0.9%), wiped using filter paper, and split longitudinally into two halves. The first part was kept at −80°C to be used for biochemical assays and gene expression. The second half was subjected to overnight fixation in paraformaldehyde (PFA 4%) solution diluted in phosphate-buffered saline (PBS) for histopathological and immunohistochemical examination.

### Oxidant/Antioxidant Hemostasis in Brain (Cerebrum and Cerebellum) Tissue Homogenates

Lipid peroxidation was determined by the formation of malondialdehyde (MDA) based on the previously-described protocol of Ohkawa et al. (1979). Tissue nitric oxide was detected following the method of Montgomery and Dymockn (Montgomery and Dymock, 1961). Tissue glutathione peroxidase (GPx) activity was estimated by the method described by Paglia and Valentine (1967), while the enzymatic activity of superoxide dismutase (SOD) was evaluated as described by Nishikimi et al. (1972).

### Butyrylcholinesterase Activity

Tissue butyrylcholinesterase activity was determined as previously described by Knedel and Böttger (1967).

### Histopathological Examination

Brain tissues (cerebrum and cerebellum) were excised and washed with PBS (pH 7.4) and subjected for overnight fixation in 4% PFA diluted in PBS. The fixed specimens were embedded in paraffin. Briefly, the tissues were dried using ascending concentrations of ethanol, cleared three times in xylene, and impregnated in melted paraffin 3 times at 65°C. Then paraffin blocks were cut into four μm thick sections and stained with either hematoxylin and eosin (H&E) as previously described by Bancroft and Layton (2013) or periodic acid Schiff (PAS) (Bancroft and Layton, 2013).

### Immunohistochemical Staining

The immunohistochemical technique of brain sections was investigated following the procedure reported by and (Mohamed et al., 2015). Briefly, the 4 μm-thick paraffin sections were prepared, deparaffinized by xylene, and rehydrated in ethanol alcohol and washed with distilled water. Afterward, endogenous peroxidase activity was deactivated by immersing the tissue sections in 3% H_2_O_2_ in absolute methanol for 30 min at room temperature and washed again using PBS. Blocking of the non-specific reaction was performed at room temperature with 10% normal blocking serum for 60 min. Then, the sections were incubated overnight at 4°C with the primary antibodies, washed with PBS, and then incubated for 60 min with biotin-conjugated goat anti-rabbit IgG antiserum or anti-mouse IgG antiserum (Histofine kit, Nichirei Corporation) according to the species’ primary antibody hosted. Then sections were washed in PBS, followed by 30 min of incubation with streptavidin-peroxidase conjugate (Histofine package, Nichirei Corporation). The streptavidin-biotin complex was set to react with a solution of 3,3’-diaminobenzidine tetrahydrochloride (DAB)-H_2_O_2_, pH 7.0 for 3 min. Finally, these sections were rinsed with distilled water and Mayer’s hematoxylin to counterstain. A digital camera (Leica EC3, Leica, Germany) connected to a microscope (Leica DM500, Leica, Germany) was used to capture the micrographs of the stained sections. Dilutions, sources, methods, and antibodies for antigen recovery were listed in Table 1.

**TABLE 1:** List of antibodies, sources, working dilutions, and methods for antigen retrieval.

Heating condition	Antigen retrieval	Dilution	Source	Antibody
105°C, 20 min	10 mM citrate buffer (pH 6.0)	1:300	(9662, Cell Signaling Technology, Danvers, Ma, United States)	Rabbit polyclonal anti-Caspase 3
105°C, 20 min	10 mM citrate buffer (pH 6.0)	1:1,200	(019-19741, Wako Osaka, Japan)	Rabbit polyclonal anti-Iba-1
105°C, 20 min	10 mM citrate buffer (pH 6.0)	1:50	(M7315, Dako, Glostrup, Denmark)	Mouse monoclonal anti-synaptophysin
105°C, 20 min	10 mM citrate buffer (pH 6.0)	1:500	(E10340, Spring Bioscience, Pleasanton, CA, United States)	Rabbit polyclonal anti-calbindin antibody

### Quantitative Reverse Transcription-Polymerase Chain Reaction (RT-qPCR)

The iNtRON biotechnology Inc RNA-spintm total RNA extraction kits (Cat. #17211) were used to extract total RNA from the brain tissues. Total RNA (1 mg) was used as a template to make the first complementary DNA (cDNA) strand using Maxima First Strand cDNA synthesis kits from iNtRON Biotechnology Inc (Cat. #EZ00SS). RT-qPCR was conducted using Thermo Scientific Maxima SYBR Green/ROX qPCR PreMix kits from iNtRON Biotechnology Inc (Cat. #RT500S) and the primers were used for neuron-specific enolase, caspase-3, glial fibrillary acidic protein, B-cell lymphoma 2 as shown in Table 2. Gene primer sequences were designed using Primer3 and BLAST programs (National Center for Biotechnology Information, Bethesda MD, 20894 United States). The targeted gene values were normalized to the expression level of the housekeeping gene GAPDH. The PCR cycle parameters were one cycle for 2 min at 50°C; one cycle for 10 min at 95°C; 40 cycles for 15 s at 95°C and for 30 s at 60°C and a final cycle for 30 s at 72°C.

**TABLE 2:** Primers sequence used for real-time PCR.

Gene primer	Accession number	Sequences
Caspase-3	NM_001284409.1	AGTTGGACCCACCTTGTGAG AGTCTGCAGCTCCTCCACAT
B-cell lymphoma 2	NM_009741.5	CACCCCTGGCATCTTCTCCTT AGCGTCTTCAGAGACAGCCAG
Glial fibrillary acidic protein	NM_017009.2	GCTGACGTTTACCAGGCAGA CCGGGCACTGTTGGTAGTAA
Neuron-specific enolase	NM_139325.4	GTACCACACACTCAAGGGG ATGGCTTCCTTCACCAGCTC

### Statistical Analysis

Data were presented as mean ± SEM. Results were statistically analyzed using a one-way ANOVA test using the Statistical Analysis System (SAS) software version 9.3 (2016). Significantly different means were compared with Tukey’s *post hoc* multiple comparison test. Results at *p* ≤ 0.05 were considered statistically significant.

## Results

### Characterization of Nanoparticles

**FIGURE 1:**
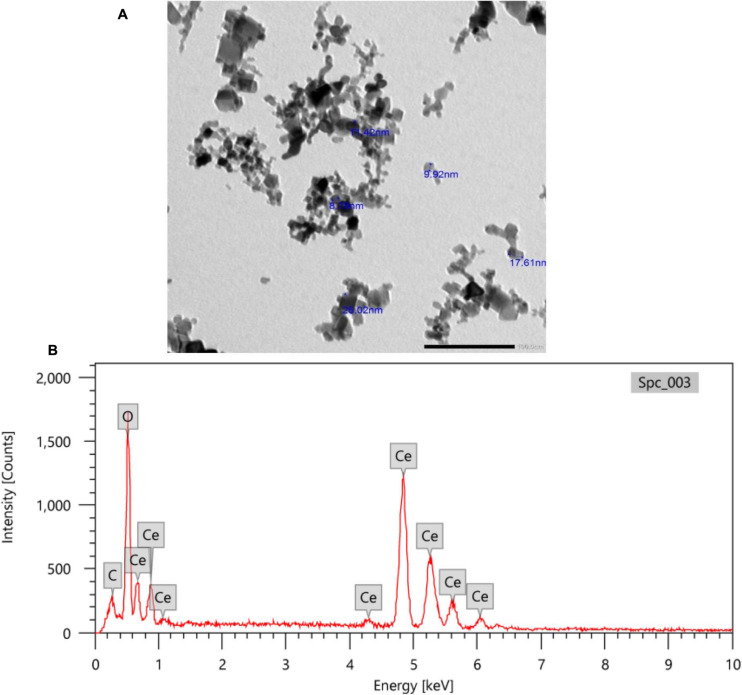
TEM imaging **(A)** and X-ray EDA pattern of CeNPs nanoparticles **(B)**.

TEM images of CeNPs showed spherical particles with sizes ranging from 9 to 25 nm (Figure 1A). Also, the X-ray EDA patterns (Figure 1B) displayed that Ce and O_2_ were the dominant (94%) atoms with few C atoms (6%).

### General Observations

No mortalities or serious clinical toxicological signs have been observed on rats exposed to sublethal doses of FIPs and/or CeNPs during the experimental period.

### Brain Lipid Peroxidation and Antioxidant Status of Brain Tissues

**FIGURE 2:**
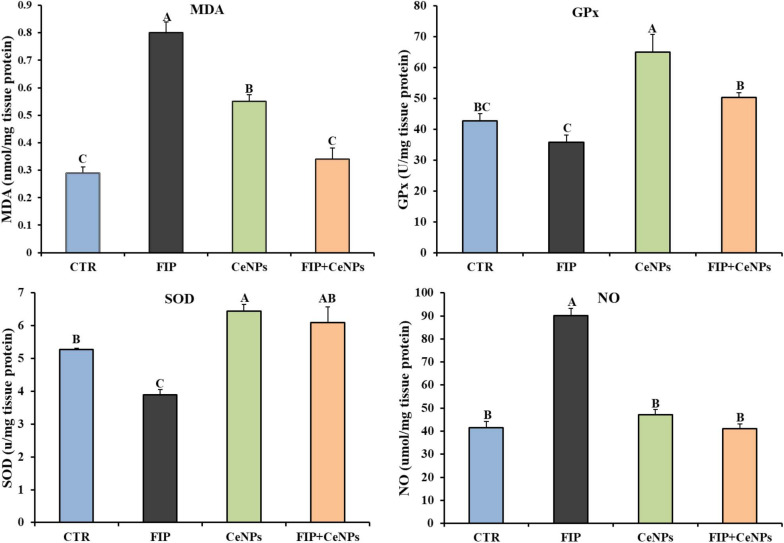
The effects of FIP, CeNPs, and FIP + CeNPs repetitive doses on lipid peroxidation and antioxidant enzyme activities of the brain tissue of rats. The data was demonstrated as mean ± SEM (*n* = 7). Means (columns) labeled with different letters **(A–D)** were significantly different at *p* ≤ 0.05). CTR, Control; FIP, Fipronil; CeNPs, cerium oxide nanoparticle; MDA, Malondialdehyde; SOD, Superoxide dismutase; GPx, Glutathione peroxidase; NO, Nitric oxide.

Compared to the control group, the FIP-intoxicated group exhibited a significant increase (*p* < 0.05) in NO and MDA levels, meanwhile rats treated with FIP + CeNPs exhibited substantial reduction (*p* < 0.05) of NO and MDA levels (Figures 2A,B) and were not significantly different from the control group. The CeNPs treatment showed increased MDA amounts compared to the control but less than the FIP treatment (Figure 2A). The FIP-intoxicated group showed a significant decrease in GPx and SOD antioxidant enzyme activities in brain tissues. On the other hand, rats treated with FIP + CeNPs showed significantly (*p* < 0.05) increased GPx and SOD enzyme activity compared to the FIP group but the levels of activity were similar to the control group (Figures 2C,D). Only the CeNPs treatment induced greater activity of GPx and SOD compared to both control and FIP-treated groups (Figures 2C,D).

### Butyrylcholinesterase

**FIGURE 3:**
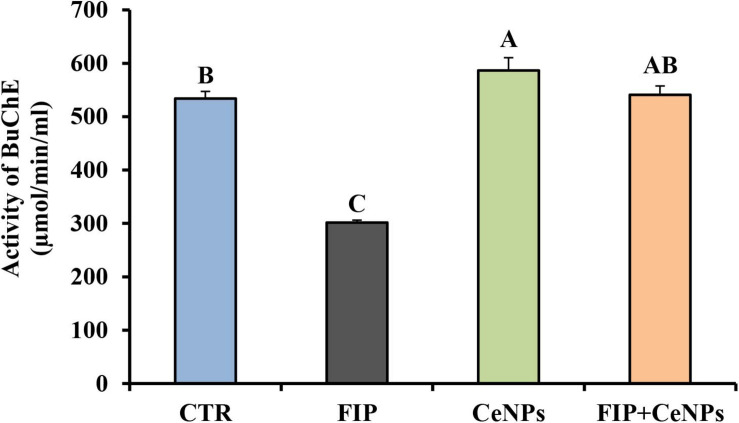
Brain butyrylcholinesterase activity (μmoL/min/mL/mg protein) in control and FIP, CeNPs and FIP + CeNPs-treated groups. Means (charts) of with different label letters **(A–D)** were varied significantly at *p* ≤ 0.05. FIP, Fipronil; CeNPs, cerium nanoparticle, and CTR: Control (*n* = 7).

The activity of butyrylcholinesterase (BuChE) in FIP-intoxicated rats has decreased significantly (*p* ≤ 0.05) in relation to the control group, while the FIP + CeNPs group showed a significant increase in the BuChE activity compared to the FIP-treated rats but not significantly different from the control rats (Figure 3). The CeNPs-treated rats showed increased BuChE activity compared to both FIP and control rats but not different from the FIP + CeNPs group.

### Histopathological Examinations

**FIGURE 4:**
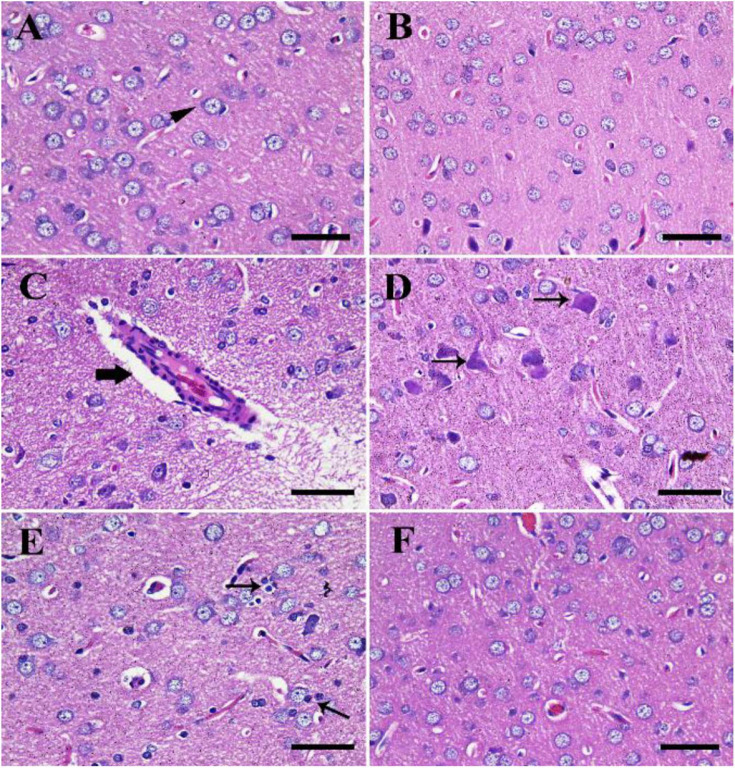
Histopathological examination of rat cerebrum. **(A)** Negative control group. **(B)** CeNPs group. **(C–E)** FIP group showing perivascular lymphocytic cuffing (thick arrow in **C**), neuronal necrosis (thin arrows in **D**), and neuronophagia (arrows in **E**). **(F)** FIP treated with CeNPs. Scale bar = 50 μm.

The negative control group showed normal cerebral architecture with normal healthy neurons (Figure 4A). Moreover, the CeNPs group did not reveal any toxic symptoms and showed cerebral architecture identical to the negative control one (Figure 4B). Whereas the FIP group showed severe vascular congestion, perivascular lymphocytic cuffing, neuronal necrosis, and satellitosis (Figures 4C–E). FIP group treated with CeNPs revealed nearly normal cerebral architecture (Figure 4F).

**FIGURE 5:**
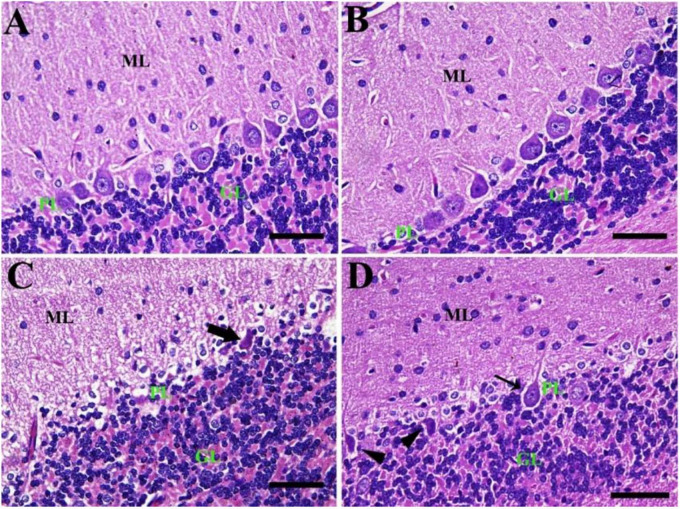
Histopathological examination of rat cerebellum. **(A)** Negative control group with the three layers of the cerebellar cortex from outside to inside; molecular layer (ML), Purkinje cell layer (PL), granule cell layer (GL). **(B)** CeNPs group. **(C)** FIP group showing shrunken Purkinje cells (arrows). **(D)** FIP treated with CeNPs. Scale bar = 50 μm.

Similarly, the negative control group reported a normal cerebellar structure with normal healthy neurons and revealed three layers of the cerebellar cortex from outside to inside; molecular layer (ML), Purkinje cell layer (PL), and granule cell layer (GL) (Figure 5A). Furthermore, the CeNPs group did not show any toxic lesion (Figure 5B). On the other hand, the FIP group revealed injured Purkinje cells manifested by shrunken cells, some cells with hyper-eosinophilic cytoplasm, loss of dendritic arborization. There was neuropil spongiosis in ML (Figure 5C). The FIP group treated with CeNPs showed nearly normal cerebellar architecture (Figure 5D).

**FIGURE 6:**
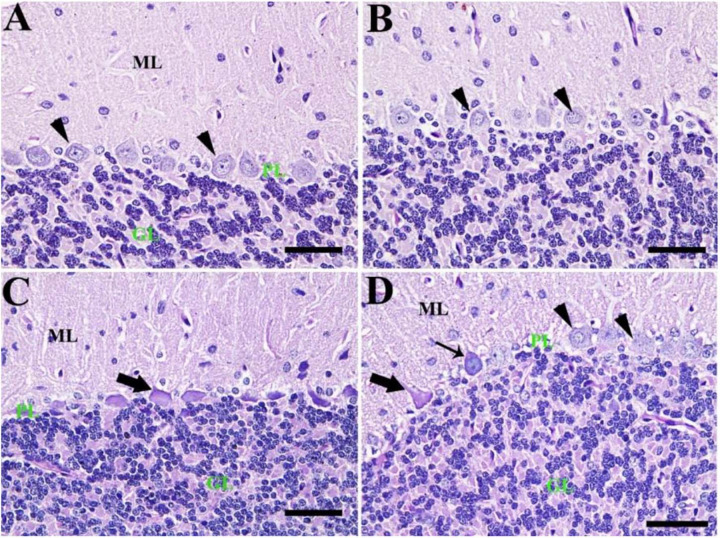
Histochemical staining of rat cerebellum by periodic acid Schiff (PAS). **(A)** Negative control group with the three layers of the cerebellar cortex from outside to inside; molecular layer (ML), Purkinje cell layer (PL), granule cell layer (GL). **(B)** CeNPs group. **(C)** FIP group showing shrunken Purkinje cells (arrows). **(D)** FIP treated with CeNPs. Normal Purkinje cells (arrowheads), necrotic Purkinje cells (thick arrows), and degenerated Purkinje cells (thin arrow). Scale bar = 50 μm.

By PAS staining, the control group showed normal cerebellar architecture normal Purkinje cells with no PAS reaction (Figure 6A). Also, the CeNPs group revealed normal Purkinje cells (Figure 6B). However, the FIP group showed shrunken Purkinje cells with a high PAS reaction (Figure 6C). FIP group treated with CeNPs revealed many normal Purkinje cells with no PAS reaction and some shrunken positive PAS Purkinje cells. Highly basophilic Purkinje cells could be detected (Figure 6D).

### Immunohistochemical Studies

**FIGURE 7:**
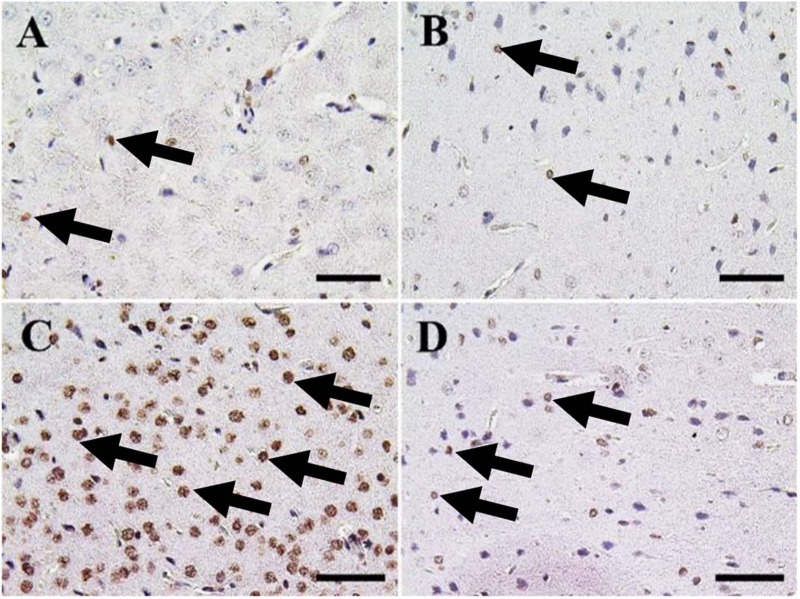
Immunohistochemical staining of rat cerebrum by caspase-3. **(A)** Negative control group. **(B)** CeNPs group. **(C)** FIP group. **(D)** FIP treated with CeNPs. Notice caspase-3 positive apoptotic neuronal cell nuclei (arrows) with numerous numbers in **(C)**, and few number in **(A,B,D)**. Scale bar = 50 μm.

The cerebrum of both negative control and CeNPs treated groups revealed few caspase-3 positive cells (Figures 7A,B). However, in the FIP group, the majority of neuronal nuclei showed positive caspase-3 reactions (Figure 7C). FIP group treated with CeNPs revealed a critical decrease in the number of caspase-3 positive neuronal cells (Figure 7D).

**FIGURE 8:**
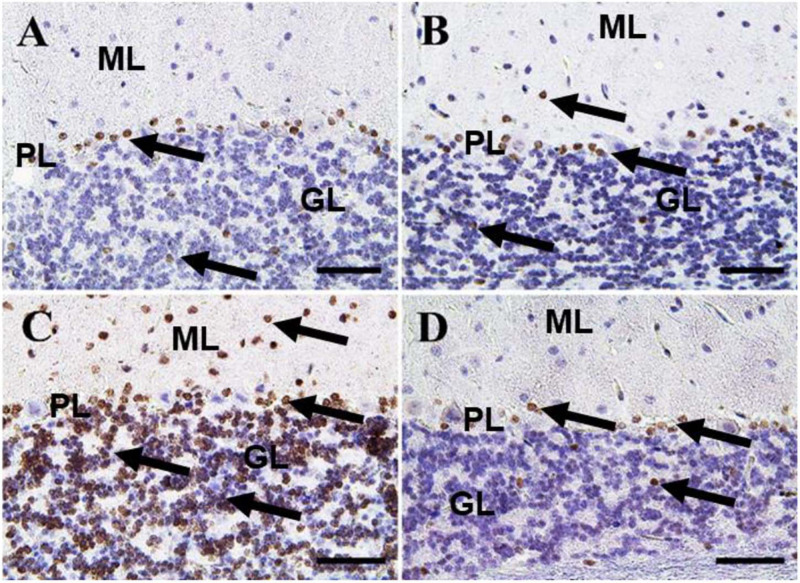
Immunohistochemical staining of rat cerebellum by caspase-3. **(A)** Negative control group. **(B)** CeNPs group. **(C)** FIP group. **(D)** FIP treated with CeNPs. Notice caspase-3 positive apoptotic neuronal cell nuclei (arrows) in all cerebellar cortex layers: molecular layer (ML), Purkinje cell layer (PL), granule cell layer (GL). Numerous number was observed in **(C)**, and few numbers in **(A,B,D)**. Scale bar = 50 μm.

In the cerebellum, negative control and CeNPs groups showed few cells with caspase-3 positive nuclei (Figures 8A,B). On the other hand, the FIP group revealed a massive positive caspase-3 reaction in all cerebellar layers (Figure 8C). Interestingly, the FIP group treated with CeNPs showed few cells with positive caspase-3 reactions (Figure 8D).

**FIGURE 9:**
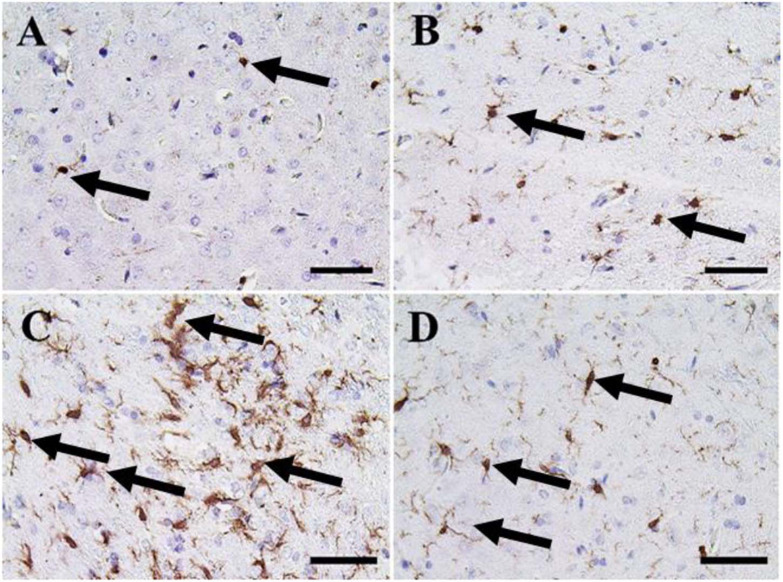
Immunohistochemical staining of rat cerebrum by ionized calcium-binding adapter molecule 1 (Iba-1). **(A)** Negative control group. **(B)** CeNPs group. **(C)** FIP group. **(D)** FIP treated with CeNPs. Notice Iba-1 positive microglia (arrows) with numerous numbers in **(C)**, and few numbers in **(A,B,D)**. Scale bar = 50 μm.

A small number of Iba-1 positive microglia were observed in the cerebrum of the negative control and CeNPs groups (Figures 9A,B). While the FIP group showed massive Iba-1 positive microglia in the cerebrum (Figure 9C). A small number of Iba-1 positive microglia were identified in the FIP group treated with CeNPs (Figure 9D).

**FIGURE 10:**
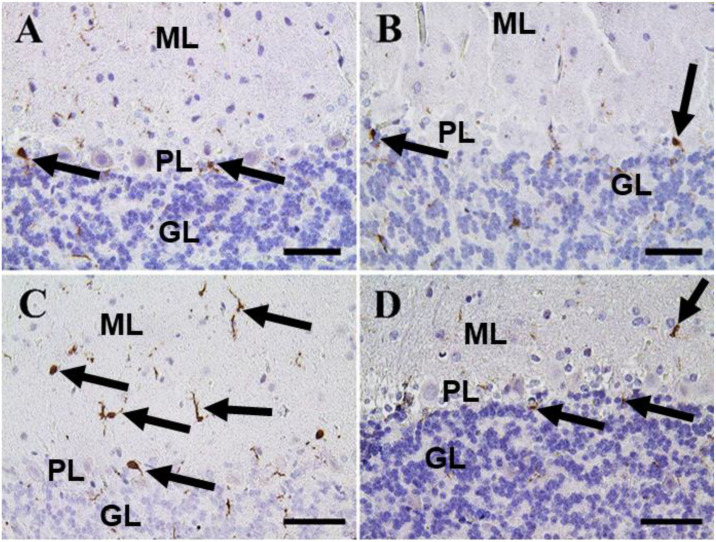
Immunohistochemical staining of rat cerebellum by Iba-1. **(A)** Negative control group. **(B)** CeNPs group. **(C)** FIP group. **(D)** FIP treated with CeNPs. Notice cerebellar cortex layers: molecular layer (ML), Purkinje cell layer (PL), granule cell layer (GL). Numerous numbers of Iba-1 positive microglia (arrows) was observed in **(C)**, and few number in **(A,B,D)**. Scale bar = 50 μm.

In the cerebellum, negative control and CeNPs groups showed a small number of positive Iba-1 microglia (Figures 10A,B). On the other hand, the FIP group revealed that Iba-1 positive microglia was widely distributed in the molecular layer and moderately distributed in the other layers of the cerebellum (Figure 10C). FIP group treated with CeNPs showed few Iba-1 positive microglia in the cerebellum (Figure 10D).

**FIGURE 11:**
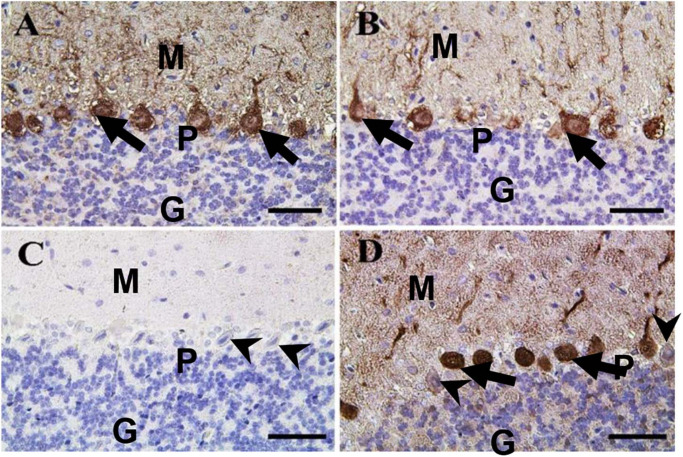
Immunohistochemical staining of rat cerebellum by calbindin. **(A)** Negative control group. **(B)** CeNPs group. **(C)** FIP group. **(D)** FIP treated with CeNPs. Notice cerebellar cortex layers: molecular layer (ML), Purkinje cell layer (PL), granule cell layer (GL). Purkinje cells showed calbindin positive reaction (arrows) in **(A,B,D)**. Negative reaction (arrow heads) was observed in all Purkinje cells in **(C)**, and some Purkinje cells in **(D)**. Scale bar = 50 μm.

In the cerebellum, calbindin showed a strong reaction in the Purkinje cells of negative control and CeNPs groups (Figures 11A,B). However, the FIP group revealed a negative calbindin reaction in all cerebellar layers (Figure 11C). The FIP group treated with CeNPs showed a strong calbindin positive reaction in most of the Purkinje cells (Figure 11D).

**FIGURE 12:**
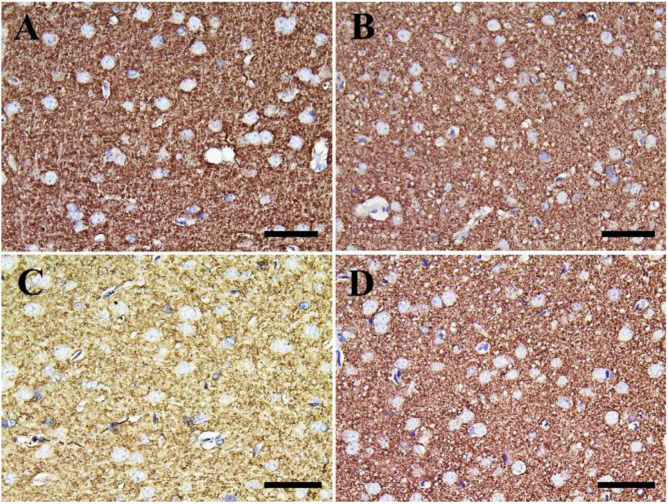
Immunohistochemical staining of rat cerebrum by synaptophysin. **(A)** Negative control group. **(B)** CeNPs group. **(C)** FIP group. **(D)** FIP treated with CeNPs. Scale bar = 50 μm.

**FIGURE 13:**
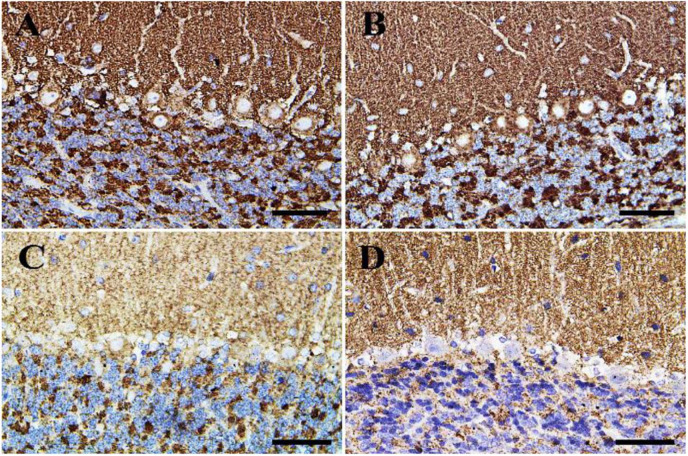
Immunohistochemical staining of rat cerebellum by synaptophysin. **(A)** Negative control group. **(B)** CeNPs group. **(C)** FIP group. **(D)** FIP treated with CeNPs. Scale bar = 50 μm.

Negative control and CeNPs groups showed strong synaptophysin reactions in the cerebrum (Figures 12A,B) and cerebellum (Figures 13A,B). On the other hand, the FIP group revealed a weak synaptophysin reaction in the cerebrum (Figure 12C) and cerebellum (Figure 13C) with a strong synaptophysin reaction in the granular layer of the cerebellum (Figure 13C). The FIP group treated with CeNPs showed a strong synaptophysin reaction in the cerebrum (Figure 12D) and cerebellum (Figure 13D).

### Quantitative Reverse Transcription-Polymerase Chain Reaction (RT-qPCR)

**FIGURE 14:**
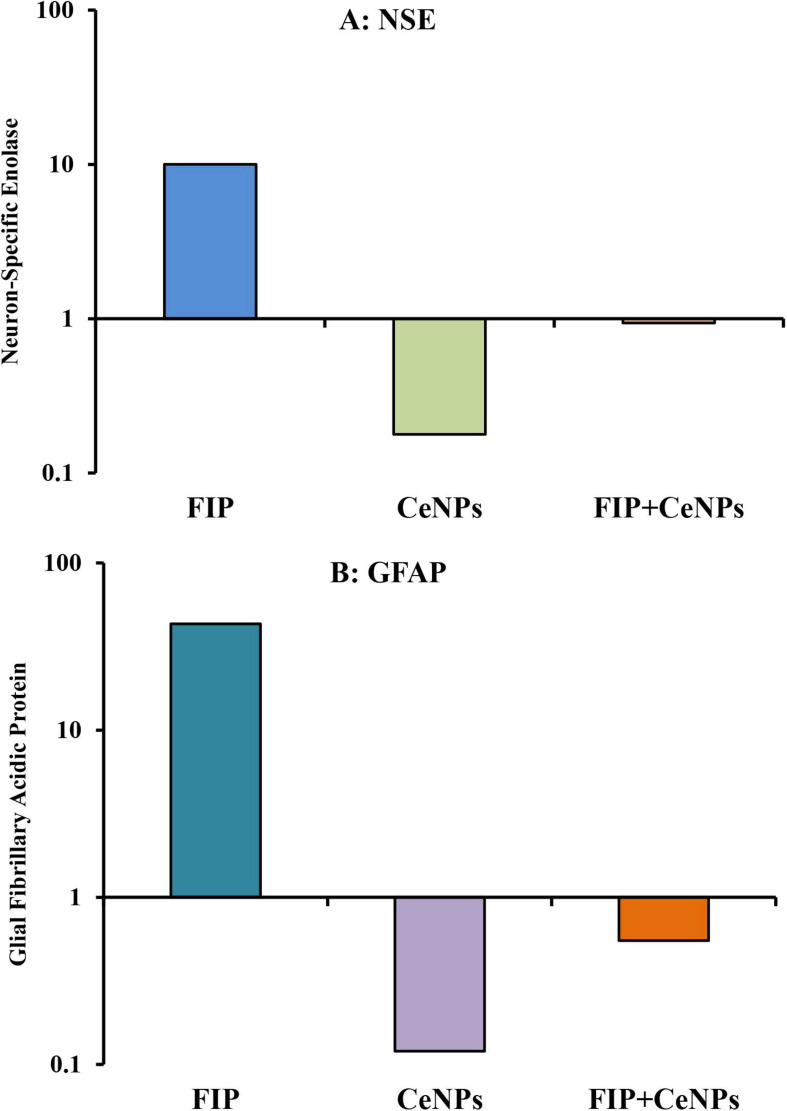
mRNA relative expression of brain function genes. **(A)** NSE, neuron-specific enolase. **(B)** GFAP, glial fibrillary acidic protein in brain tissue of male albino rat exposed to FIP and intoxicated with CeNPs. The expression of transcripts was normalized to GAPDH. The data is demonstrated as mean ± SEM (*n* = 7). FIP, Fipronil; CeNPs, cerium nanoparticle.

**FIGURE 15:**
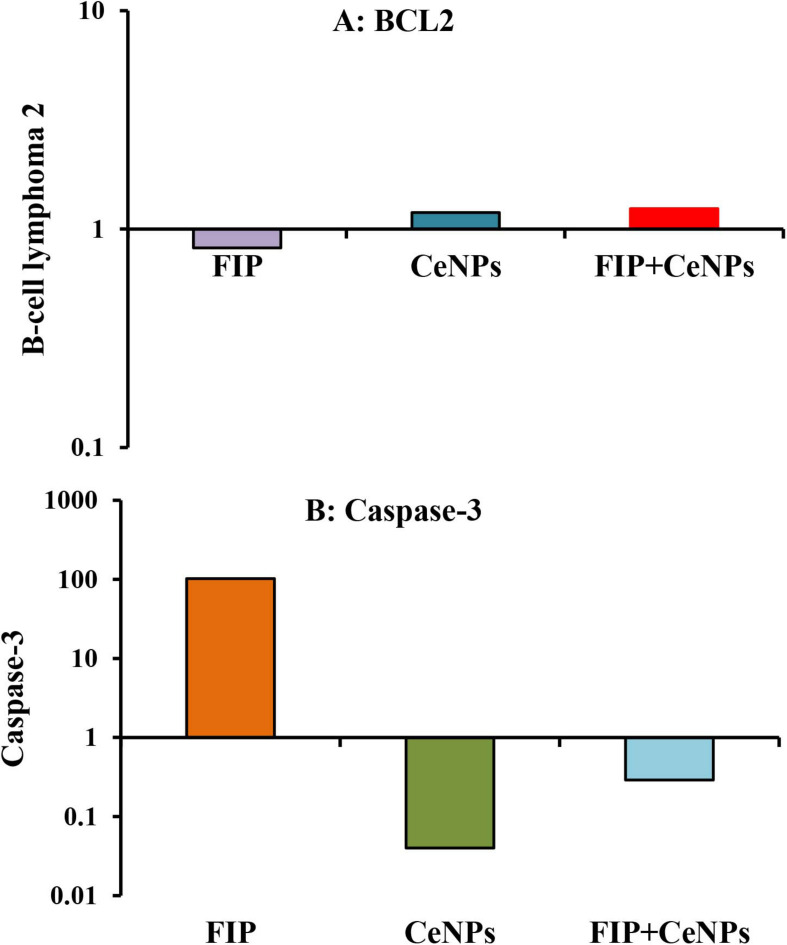
mRNA relative expression of apoptosis-related genes **(A)** BCL-2 and **(B)** caspase-3 in the brain tissues of male albino rats exposed to FIP and intoxicated with CeNPs. The expression of transcripts was normalized to GAPDH. The data is demonstrated as mean ± SEM (*n* = 7). FIP, Fipronil; CeNPs, cerium nanoparticle.

The results showed that the relative mRNA expressions of glial fibrillary acidic protein (GFAP) and neuron-specific enolase (NSE) (Figures 14A,B) and caspase-3 (Figure 15A) were significantly up-regulated (*p* ≤ 0.05). The BCL-2 gene was down-regulated in the brain tissue of rats that received FIP when compared to the control. While in the case of the FIP group treated with CeNPs, significantly (*p* ≤ 0.05) down-regulated the GFAP, NSE (Figures 14A,B), and caspase-3 (Figure 15A) and up-regulated BCL-2 (Figure 15B).

## Discussion

FIP, a phenylpyrazole compound, induces hepatotoxicity, neurotoxicity spermatotoxicity, and growth retardation (Ki et al., 2012; Gupta et al., 2013; Badgujar et al., 2015; Khan et al., 2015). Because of the wide commercial and domestic uses of FIP, concerns about its adverse effects on public health have been raised. Although phenyl pyrazole neurotoxicity is well-characterized, and their mechanism of action in mammals is already known. However, publication on the potential neurobehavioral effect of this class of insecticides on mammals is limited (Kanat and Selmanoğlu, 2020). Lipid peroxidation (LPO) requires polyunsaturated fatty acid (PUFA) oxidative deterioration that alters the membrane structure and functions, which warrant the reduction of membrane fluidity and inactivation of membrane-bound enzymes (Gutteridge and Halliwell, 2000). Based on results reported herein and in the literature, there is an increasing evidence that FIP might cause a variety of toxic effects to animals and humans, such as neurotoxic, hepatotoxic, nephrotoxic, reproductive, and cytotoxic effects on vertebrate and invertebrates. In the last decade, oxidative stress has been suggested to be involved in the various toxicities induced by FIP (Wang et al., 2016). FIP was cytotoxic to these cells and its cytotoxicity showed a concentration-dependent manner (Lee et al., 2011). Neuronal cell death caused by FIP was, also, attributed to ROS generation and oxidative stress (Ki et al., 2012). It has been documented that both FIP (100 μM) and FIP sulfone (37 μM) treatments substantially increased the NO production in SH-SY5Y cells, suggesting that oxidative stress might be one of the main mechanisms of the neurotoxicity of FIP (Romero et al., 2016). Additionally, the high level of LPO in rats treated with FIP was attributed to increased ROS production, mainly the hydroxyl radicals, which could damage the antioxidant protection system (Banerjee et al., 1999). Subsequently, the ROS generation might affect the mitochondrial function and lipid peroxidation (MDA) levels, leading to cell injury (Chen et al., 2010).

The reduction of the SOD enzyme activity in animals treated with FIP reported in our study might be attributed to its use as an antioxidant to convert the free radical formed O_2_ to H_2_O (Badgujar et al., 2016; Kartheek and David, 2018). The SOD and GPx are known as the first protection mechanism to defend cells from oxidative stress caused by ROS (Ighodaro and Akinloye, 2018). FIP intoxicated rats showed increased concentrations of MDA and NO. Subsequently, FIP caused alteration in antioxidant enzymes by SOD and GPx that might initiate damage to cellular macromolecules, including proteins, lipids, and DNA (Antunes et al., 2002; Clasen et al., 2012; Khan et al., 2015; Weidinger and Kozlov, 2015). The present data showed that FIP exposure induced an extreme reduction in both SOD and GPx levels in brain tissue, which might be due to excessive production of O^2–^ (Mossa et al., 2015). Along with that, the present study demonstrated a possible neuroprotective effect of CeNPs through its antioxidant activity. Because of its reported ability to pass the blood-brain barrier (BBB) making it a suitable prospect for the neural diseases’ treatment (Rzigalinski et al., 2017; Song et al., 2020) in the case of FIP intoxicated rats. Also, CeNPs showed increased activity of antioxidant enzymes (SOD and GPx) with lower levels of the biomarkers of lipid peroxidation (MDA and NO). CeNPs antioxidant properties might be due to its ability to transform from oxidized to reduced form (Ce^3+^ and Ce^4+^) and vice versa found on the surface (Karakoti et al., 2010). The ratio of Ce^3+^/Ce^4+^ on the surface of nanoparticles was reported to be significantly related to the activity of redox as it could activate the scavenging process of both reactive nitrogen species (RNS) and ROS in animals (Estevez et al., 2011).

Nanoceria has been able to exhibit its nitric oxide radical scavenging ability, which is coined by the presence of nanoparticles with a low ratio of Ce^3+^/Ce^4+^ (Dowding et al., 2012). Also, nanomaterials might interact with different types of ROS, particularly with O^2–^ and H_2_O_2_, and had so-called catalase (CAT-) and SOD-mimetic activities (Heckert et al., 2008; Pirmohamed et al., 2010; Zhou et al., 2016). Thus, the prospective protective effects of CeNPs neurotoxicity are influenced by suppressing apoptosis and oxidative stress possibly by its antioxidant properties (Ghaznavi et al., 2015). Both inflammatory responses and oxidative stress have been detected as essential elements that initiate neuronal cell injury (Hunot et al., 1997; Slemmer et al., 2008). Ionized calcium-binding adaptor molecule 1 (Iba-1) is a cytoplasmic protein known to be a pan microglial marker (Walker and Lue, 2015) and expressed primarily in brain microglia, indicating that it plays a significant role in controlling microglia function (Imai et al., 1996; Hirasawa et al., 2005). Previous studies have shown its associated expression with microglial inflammation and activation (Ito et al., 1998; Streit et al., 2009; Minett et al., 2016). The present study revealed that FIP-intoxicated rats caused massive Iba-1 positive microglia distribution in the cerebrum and the molecular layer with moderate distribution in the other layers of the cerebellum. On the other hand, the CeNPs reduced these inflammations, so it could protect neurons from damage that approved by a low number of Iba-1 positive microglia and moderate Iba-1 microglia in the cerebellum in FIP + CeNPs-treated group.

In previous studies, a strong association was found between the glial functions biomarkers and inflammation and BuChE (Darreh-Shori et al., 2013). They suggested that functional variability in BuChE activity, depend on allelic variation in the BuChE gene that regulates the intrathecal astroglial biomarker profile and cytokines. Thus, reduced BuChE enzymatic activity, either because of genetic K variant protein or phenotypic modulation by the apolipoprotein E (ApoE), is associated with worse cognitive performance and *in vivo* pathological signs (Darreh-Shori et al., 2012). BuChE activity plays an important role in regulating intrinsic inflammation and activity of cholinoceptive glial cells and that this might be of clinical relevance. The dissociation between astroglial markers and inflammatory cytokines indicates that a proper activation and maintenance of astroglial function is a beneficial response, rather than a disease-driving mechanism (Darreh-Shori et al., 2013).

Earlier studies have established that acetylcholine (ACh), the classical neurotransmitter in the central and peripheral nervous systems, acts as a suppressor of inflammatory responses of lymphocytes, mediated by binding to α7-nicotinic ACh receptors (α7-nAChRs) (Parrish et al., 2008). This is known as the cholinergic anti-inflammatory pathway, by which the nervous system is proposed to exert immunomodulatory effects on systemic immunity (Pavlov et al., 2009). It has been reported that synaptically released ACh could also be hydrolyzed to choline and acetate by glial BuChE, in a manner analogous to the inactivation of glutamate in glutamatergic transmission (Mesulam et al., 2002). Neurological effects were identified after exposure to pesticides, which inhibit the BuChE activity (Rohlman et al., 2011). Results reported in the present study indicated that BuChE inhibition was a significant indicator of FIP exposure.

Moreover, glial fibrillary acidic protein (GFAP) an astrogliosis biomarker is a cellular reaction that indicates both glial and neuronal injuries (Roberts et al., 2015). Only nestin-positive stromal cells are able to differentiate into GFAP-positive cells when they are co-cultivated with neural stem cells. A proposal that adult neural progenitors express the intermediate filament GFAP and share ultra-structure characteristics with astro glia (Garcia et al., 2004). GFAP was believed to occur in non-myelinating Schwann cells in the peripheral nervous system (PNS), and the enteric glial cells in the enteric nervous system (ENS) (Laranjeira et al., 2011; Gulbransen and Sharkey, 2012), which might be a suitable measure for patients with brain injury (Schiff et al., 2012). Based on the superior function of CeNPs as a regenerative antioxidant, it would be reasonable to proceed in the application of CeNPs to cure neurodegenerative disorders (Rzigalinski et al., 2017). In the present study, upregulation of GFAP in case of FIP intoxication, while down-regulation in case of FIP + CeNPs, treated rats were reported.

FIP induced accumulation of GABA at the synaptic junctions (Gunasekara et al., 2007). These alterations in GABAergic neurons were revealed with substantial decreases in presynaptic proteins (Stanley, 1997; Catterall and Few, 2008). Synaptophysin is a synaptic vesicle transmembrane glycoproteins necessary for neurotransmission (Disdier et al., 2017). It is one of the most commonly used protein indicators of synaptic plasticity in the brain (Reddy et al., 2005; Liu et al., 2019). It is a common and responsive synaptic terminal marker (Carvalho-Netto et al., 2011), which participates in the formation and availability of synaptic vesicle (Kwon and Chapman, 2011). Data reported herein showed weak synaptophysin reaction in the cerebrum of FIP-intoxicated rats, while FIP + CeNPs group had a strong synaptophysin reaction. Also, neuron-specific enolase (NSE) is a biochemical indicator for assessing neuronal injury in brain lesions (Sahu et al., 2017). NSE is the most acidic brain isoenzyme of the glycolytic enzyme enolase (EC4.2.1.11) and has been shown to be homologous to the 14-3-2 protein isolated from bovine brain by Moore’-3. Whereas NSE is exclusively localized in neurons in mammalian nervous tissue (Schmechel et al., 1978). High concentration of NSE was considered as an oxidative damage marker (Chaves et al., 2010; Ciancarelli et al., 2015). The present study documented that FIP-treated rats induced up-regulation of NSE that might be due to the generation of oxidative stress induced by FIP in rats’ brains, meanwhile, CeNPs has a neuroprotective effect by downregulation of NSE in FIP + CeNPs treated.

The induction of oxidative stress could alter the neuron physiological functions including signal transduction through Ca^2+^ homeostasis changes (Kheradpezhouh et al., 2016). Several vertebrate CNS neurons expressed the Ca^2+^ binding protein calbindin D-28k (CB) as one of the main calcium-binding and buffering proteins, which plays a critical role in preserving calcium homeostasis and inhibiting a neuronal death (Kook et al., 2014). It had been discussed in previous studies that CB is a crucial factor in regulating synaptic Ca^2+^ dynamics and possibly with a significant role in the plasticity and information processes. CB participates in Ca^2+^ buffered transport in neurons (Schmidt, 2012). The present data stated that, by immunohistochemical staining of rat cerebellum, calbindin in FIP-intoxicated group showed negative calbindin reaction in all cerebellar layers compared to FIP + CeNPs showed strong calbindin reaction in most of Purkinje cells which is critical evidence for neuron dysfunction caused by FIP. Also, mitochondrial Ca^2+^ overload is one of the pro-apoptotic forms of inducing mitochondrial swelling with disruption or breakup of the outer membrane and, in effect, of releasing of mitochondrial apoptotic factors into the cytosol (Rizzuto et al., 2008).

An elevated level of NO can also be synthesized by neurons or activated glial cells, reflecting on the LPO process and increasing the MDA, which exhibits neurotoxicity and causes apoptotic cell death and therefore induce neuronal damage in various neuronal cells (Liu et al., 2010). Also, FIP could induce cell death by decreasing pro-inflammatory factors associated with MAPK (Park et al., 2016b). The combination of mitochondrial Ca^2+^ and ROS production leads to mitochondrial permeability transition pore (MPTP) opening that enables proapoptotic molecules translocation from mitochondria to cytosol. MPTP activation provides an open channel that allows the free diffusion of cytochrome C release from mitochondria to the cytoplasm where caspase-9 is activated (Emerit et al., 2004; Lavrik et al., 2005). Caspases are cysteine proteases family play a major function in apoptosis modulation. After death, stimulation signals were obtained from a receptor located on the cell membrane, the initial caspase is stimulated through the extrinsic and intrinsic (mitochondrial) pathway, thus, the executed caspase is triggered to degrade and induce apoptosis of the appropriate substrate (Fuchs and Steller, 2011; Gandhi and Abramov, 2012; Zhang et al., 2015). Besides, the BCL-2 family members including Bax and BCL-2 are significant controls of mitochondrial integrity and mitochondria-initiated release of cytochrome C and caspase stimulation (Lee et al., 2011). Mitochondrial injury causes translocation of Bax from the cytosol to mitochondria, while reducing BCL-2 expression in the mitochondria (Park et al., 2016a). Similarly, FIP induces lowered BCL-2 expression levels and activated the expression of caspase-3. The CeNPs inhibited the programmed cell death pathway that may be due to its effect on the production of free radicals. Also, the CeNPs could prevent programmed cell death by regulating BCL-2, caspase-3 proteins, and Bax (Ghaznavi et al., 2015) that was indicated in the present study by increase expression of BCL-2 with decrease caspase-3 expression in the case of FIP + CeNPs in comparison to FIP exposed rats.

## Conclusion

Brain tissues of male albino rats showed that exposure to FIP insecticide induces neurotoxic effects. FIP caused oxidative stress by overproduction of ROS through increased MDA and NO levels, imbalance (decreasing) of SOD and GPx activity. FIP treatment caused significant histopathological changes in brain tissues and finally cause apoptosis by modifying the mRNA expression of BCL-2 and caspase-3. On the other hand, CeNPs ameliorated the neurotoxicity induced by FIP by scavenging of ROS involving a decrease of MDA and NO, enhancing antioxidant enzyme activity as SOD and GPx, and normalizing the mRNA expression of brain function genes. Therefore, it could be concluded that cerium nanoparticles have a neuroprotective role through antioxidant and anti-apoptotic activity.

## Data Availability Statement

All data presented in current article is found in the text.

## Ethics Statement

The animal study was reviewed and approved by the Damanhour University. The research proposal has been reviewed and approved by the Animal Health Care (AHC) Committee with the principal investigator HSh Approval no.: DMU-2022-003 and Approval period: 01/04/2019-30/03/2022.

## Author Contributions

NE, ANa, YE-S, and HSh contributed to experiment protocol and lab work. DS, ANa, LW, HSa, YE, AS, and MT contributed to data analysis. GB, ANa, MU, and HSh contributed to supervision and writing the manuscript. All authors reviewed the manuscript.

## Conflict of Interest

The authors declare that the research was conducted in the absence of any commercial or financial relationships that could be construed as a potential conflict of interest.
